# Magnetic control of tokamak plasmas through deep reinforcement learning

**DOI:** 10.1038/s41586-021-04301-9

**Published:** 2022-02-16

**Authors:** Jonas Degrave, Federico Felici, Jonas Buchli, Michael Neunert, Brendan Tracey, Francesco Carpanese, Timo Ewalds, Roland Hafner, Abbas Abdolmaleki, Diego de las Casas, Craig Donner, Leslie Fritz, Cristian Galperti, Andrea Huber, James Keeling, Maria Tsimpoukelli, Jackie Kay, Antoine Merle, Jean-Marc Moret, Seb Noury, Federico Pesamosca, David Pfau, Olivier Sauter, Cristian Sommariva, Stefano Coda, Basil Duval, Ambrogio Fasoli, Pushmeet Kohli, Koray Kavukcuoglu, Demis Hassabis, Martin Riedmiller

**Affiliations:** 1 DeepMind, London, UK; 2 Swiss Plasma Center - EPFL, Lausanne, Switzerland

**Keywords:** Nuclear fusion and fission, Computer science, Magnetically confined plasmas

## Abstract

Nuclear fusion using magnetic confinement, in particular in the tokamak configuration, is a promising path towards sustainable energy. A core challenge is to shape and maintain a high-temperature plasma within the tokamak vessel. This requires high-dimensional, high-frequency, closed-loop control using magnetic actuator coils, further complicated by the diverse requirements across a wide range of plasma configurations. In this work, we introduce a previously undescribed architecture for tokamak magnetic controller design that autonomously learns to command the full set of control coils. This architecture meets control objectives specified at a high level, at the same time satisfying physical and operational constraints. This approach has unprecedented flexibility and generality in problem specification and yields a notable reduction in design effort to produce new plasma configurations. We successfully produce and control a diverse set of plasma configurations on the Tokamak à Configuration Variable^1,2^, including elongated, conventional shapes, as well as advanced configurations, such as negative triangularity and ‘snowflake’ configurations. Our approach achieves accurate tracking of the location, current and shape for these configurations. We also demonstrate sustained ‘droplets’ on TCV, in which two separate plasmas are maintained simultaneously within the vessel. This represents a notable advance for tokamak feedback control, showing the potential of reinforcement learning to accelerate research in the fusion domain, and is one of the most challenging real-world systems to which reinforcement learning has been applied.

## Abstract (web summary)

A newly designed control architecture uses deep reinforcement learning to learn to command the coils of a tokamak, and successfully stabilizes a wide variety of fusion plasma configurations.

## Main

Tokamaks are torus-shaped devices for nuclear fusion research and are a leading candidate for the generation of sustainable electric power. A main direction of research is to study the effects of shaping the distribution of the plasma into different configurations^3–5^ to optimize the stability, confinement and energy exhaust, and, in particular, to inform the first burning-plasma experiment, ITER. Confining each configuration within the tokamak requires designing a feedback controller that can manipulate the magnetic field^6^ through precise control of several coils that are magnetically coupled to the plasma to achieve the desired plasma current, position and shape, a problem known as the tokamak magnetic control problem.

The conventional approach to this time-varying, non-linear, multivariate control problem is to first solve an inverse problem to precompute a set of feedforward coil currents and voltages^7,8^. Then, a set of independent, single-input single-output PID controllers is designed to stabilize the plasma vertical position and control the radial position and plasma current, all of which must be designed to not mutually interfere^6^. Most control architectures are further augmented by an outer control loop for the plasma shape, which involves implementing a real-time estimate of the plasma equilibrium^9,10^ to modulate the feedforward coil currents^8^. The controllers are designed on the basis of linearized model dynamics, and gain scheduling is required to track time-varying control targets. Although these controllers are usually effective, they require substantial engineering effort, design effort and expertise whenever the target plasma configuration is changed, together with complex, real-time calculations for equilibrium estimation.

A radically new approach to controller design is made possible by using reinforcement learning (RL) to generate non-linear feedback controllers. The RL approach, already used successfully in several challenging applications in other domains^11–13^, enables intuitive setting of performance objectives, shifting the focus towards what should be achieved, rather than how. Furthermore, RL greatly simplifies the control system. A single computationally inexpensive controller replaces the nested control architecture, and an internalized state reconstruction removes the requirement for independent equilibrium reconstruction. These combined benefits reduce the controller development cycle and accelerate the study of alternative plasma configurations. Indeed, artificial intelligence has recently been identified as a ‘Priority Research Opportunity’ for fusion control^14^, building on demonstrated successes in reconstructing plasma-shape parameters^15,16^, accelerating simulations using surrogate models^17,18^ and detecting impending plasma disruptions^19^. RL has not, however, been used for magnetic controller design, which is challenging due to high-dimensional measurements and actuation, long time horizons, rapid instability growth rates and the need to infer the plasma shape through indirect measurements.

In this work, we present an RL-designed magnetic controller and experimentally verify its performance on a tokamak. The control policies are learned through interaction with a tokamak simulator and are shown to be directly capable of tokamak magnetic control on hardware, successfully bridging the ‘sim-to-real’ gap. This enables a fundamental shift from engineering-driven control of a pre-designed state to artificial-intelligence-driven optimization of objectives specified by an operator. We demonstrate the effectiveness of our controllers in experiments carried out on the Tokamak à Configuration Variable (TCV)^1,2^, in which we demonstrate control of a variety of plasma shapes, including elongated ones, such as those foreseen in ITER, as well as advanced configurations, such as negative triangularity and ‘snowflake’ plasmas. Additionally, we demonstrate a sustained configuration in which two separate plasma ‘droplets’ are simultaneously maintained within the vessel. Tokamak magnetic control is one of the most complex real-world systems to which RL has been applied. This is a promising new direction for plasma controller design, with the potential to accelerate fusion science, explore new configurations and aid in future tokamak development.

## Learning control and training architecture

**Fig. 1:**
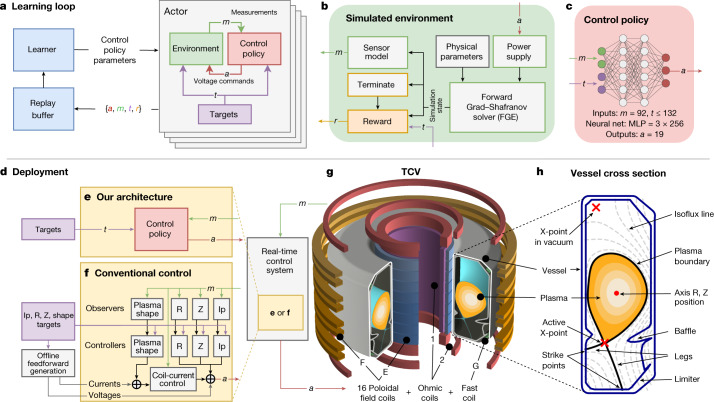
Representation of the components of our controller design architecture. **a**, Depiction of the learning loop. The controller sends voltage commands on the basis of the current plasma state and control targets. These data are sent to the replay buffer, which feeds data to the learner to update the policy. **b**, Our environment interaction loop, consisting of a power supply model, sensing model, environment physical parameter variation and reward computation. **c**, Our control policy is an MLP with three hidden layers that takes measurements and control targets and outputs voltage commands. **d**–**f**, The interaction of TCV and the real-time-deployed control system implemented using either a conventional controller composed of many subcomponents (**f**) or our architecture using a single deep neural network to control all 19 coils directly (**e**). **g**, A depiction of TCV and the 19 actuated coils. The vessel is 1.5 m high, with minor radius 0.88 m and vessel half-width 0.26 m. **h**, A cross section of the vessel and plasma, with the important aspects labelled.

Our architecture, depicted in Fig. 1, is a flexible approach for designing tokamak magnetic confinement controllers. The approach has three main phases. First, a designer specifies objectives for the experiment, potentially accompanied by time-varying control targets. Second, a deep RL algorithm interacts with a tokamak simulator to find a near-optimal control policy to meet the specified goals. Third, the control policy, represented as a neural network, is run directly (‘zero shot’) on tokamak hardware in real time.

In the first phase, the experimental goal is specified by a set of objectives that can contain a wide variety of desired properties (Extended Data Table 4). These properties range from basic stabilization of position and plasma current to sophisticated combinations of several time-varying targets, including a precise shape outline with specified elongation, triangularity and X-point location. These objectives are then combined into a ‘reward function’ that assigns a scalar quality measure to the state at each time step. This function also penalizes the control policy for reaching undesired terminal states, as discussed below. Crucially, a well-designed reward function will be minimally specified, giving the learning algorithm maximum flexibility to attain the desired outcome.

In the second phase, a high-performance RL algorithm collects data and finds a control policy through interaction with an environment, as depicted in Fig. 1a, b. We use a simulator that has enough physical fidelity to describe the evolution of plasma shape and current, while remaining sufficiently computationally cheap for learning. Specifically, we model the dynamics governing the evolution of the plasma state under the influence of the poloidal field coil voltages using a free-boundary plasma-evolution model^20^. In this model, the currents in the coils and passive conductors evolve under the influence of externally applied voltages from the power supplies, as well as induced voltages from time-varying currents in other conductors and in the plasma itself. The plasma is, in turn, modelled by the Grad–Shafranov equation^21^, which results from the balance between the Lorentz force and the pressure gradient inside the plasma on the timescales of interest. The evolution of total plasma current *I*_p_ is modelled using a lumped-circuit equation. This set of equations is solved numerically by the FGE software package^22^.

The RL algorithm uses the collected simulator data to find a near-optimal policy with respect to the specified reward function. The data rate of our simulator is markedly slower than that of a typical RL environment due to the computational requirements of evolving the plasma state. We overcome the paucity of data by optimizing the policy using maximum a posteriori policy optimization (MPO)^23^, an actor-critic algorithm. MPO supports data collection across distributed parallel streams and learns in a data-efficient way. We additionally exploit the asymmetry inherent to the actor-critic design of MPO to overcome the constraints of magnetic control. In actor-critic algorithms, the ‘critic’ learns the discounted expected future reward for various actions using the available data and the ‘actor’ uses the predictions of the critic to set the control policy. The representation of the control policy of the actor is restricted, as it must run on TCV with real-time guarantees, whereas the critic is unrestricted, as it is only used during training. We therefore use a fast, four-layer feedforward neural network in the actor (Fig. 1c) and a much larger recurrent neural network in the critic. This asymmetry enables the critic to infer the underlying state from measurements, deal with complex state-transition dynamics over different timescales and assess the influence of system measurement and action delays. The information from the coupled dynamics is then distilled into a real-time-capable controller.

In the third phase, the control policy is bundled with the associated experiment control targets into an executable using a compiler tailored towards real-time control at 10 kHz that minimizes dependencies and eliminates unnecessary computations. This executable is loaded by the TCV control framework^24^ (Fig. 1d). Each experiment begins with standard plasma-formation procedures, in which a traditional controller maintains the location of the plasma and total current. At a prespecified time, termed the ‘handover’, control is switched to our control policy, which then actuates the 19 TCV control coils to transform the plasma shape and current to the desired targets. Experiments are executed without further tuning of the control-policy network weights after training, in other words, there is ‘zero-shot’ transfer from simulation to hardware.

The control policies reliably transfer onto TCV through several key attributes of the learning procedure, depicted in Fig. 1b. We identified an actuator and sensor model that incorporates properties affecting control stability, such as delays, measurement noise and control-voltage offsets. We applied targeted parameter variation during training across an appropriate range for the plasma pressure, current density profile and plasma resistivity through analysis of experiment data, to account for varying, uncontrolled experimental conditions. This provides robustness while ensuring performance. Although the simulator is generally accurate, there are known regions where the dynamics are known to be poorly represented. We built ‘learned-region avoidance’ into the training loop to avoid these regimes through the use of rewards and termination conditions (Extended Data Table 5), which halt the simulation when specified conditions are encountered. Termination conditions are also used to enforce operational limits. The control policies learn to stay within the specified limits, for example, on maximum coil current or the edge safety factor^25^.

The controllers designed by our architecture are greatly structurally simplified compared with conventional designs, as depicted in Fig. 1e, f. Instead of a series of controllers, RL-driven design creates a single network controller.

## Fundamental capability demonstration

We demonstrate the capability of our architecture on control targets in real-world experiments on TCV. We first show accurate control of the fundamental qualities of plasma equilibria. We then control a wide range of equilibria with complex, time-varying objectives and physically relevant plasma configurations. Finally, we demonstrate control of a configuration with several plasma ‘droplets’ in the vessel simultaneously.

We first test the fundamental tasks of plasma control through a series of changes representative of those required for a full plasma discharge. First, from the handover at 0.0872 s, take over and stabilize *I*_p_ at −110 kA. Next, ramp the plasma current to −150 kA and then elongate the plasma from 1.24 to 1.44, thereby increasing the vertical instability growth rate to 150 Hz. Next, demonstrate position control through shifting the vertical plasma position by 10 cm and then divert the plasma with control of the active X-point location (see Fig. 1h). Finally, return the plasma to the handover condition and ramp down *I*_p_ to −70 kA to shut down safely. Although accuracy requirements will generally depend on the exact experiment, a reasonable aim is to control *I*_p_ to within 5 kA (3% of the final 150-kA target) and the shape to within 2 cm (8% of the vessel radial half width of 26 cm). Note that the equilibrium reconstruction used matches a visually reconstructed boundary with a typical accuracy^26^ of 1 cm.

**Fig. 2:**
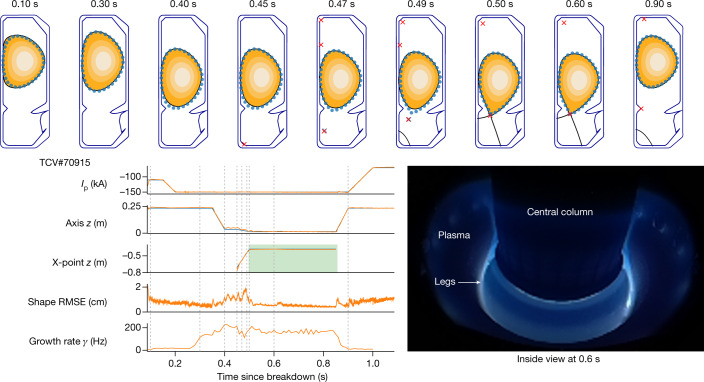
Fundamental capability demonstration. Demonstration of plasma current, vertical stability, position and shape control. Top, target shape points with 2 cm radius (blue circles), compared with the post-experiment equilibrium reconstruction (black continuous line in contour plot). Bottom left, target time traces (blue traces) compared with reconstructed observation (orange traces), with the window of diverted plasma marked (green rectangle). Bottom right, picture inside the vessel at 0.6 s showing the diverted plasma with its legs. Source data

The performance of the control policy is depicted in Fig. 2. All tasks are performed successfully, with a tracking accuracy below the desired thresholds. In the initial limited phase (0.1 s to 0.45 s), the *I*_p_ root-mean-square error (RMSE) is 0.71 kA (0.59% of the target) and the shape RMSE is 0.78 cm (3% of the vessel half width). In the diverted phase (0.55 s to 0.8 s), the *I*_p_ and shape RMSE are 0.28 kA and 0.53 cm, respectively (0.2% and 2.1%), yielding RMSE across the full window (0.1 s to 1.0 s) of 0.62 kA and 0.75 cm (0.47% and 2.9%). This demonstrates that our RL architecture is capable of accurate plasma control across all relevant phases of a discharge experiment.

## Control demonstrations

**Fig. 3:**
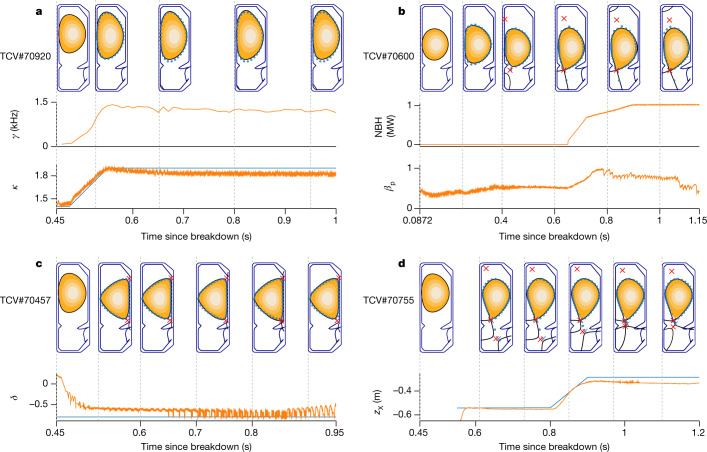
Control demonstrations. Control demonstrations obtained during TCV experiments. Target shape points with 2 cm radius (blue circles), compared with the equilibrium reconstruction plasma boundary (black continuous line). In all figures, the first time slice shows the handover condition. **a**, Elongation of 1.9 with vertical instability growth rate of 1.4 kHz. **b**, Approximate ITER-proposed shape with neutral beam heating (NBH) entering H-mode. **c**, Diverted negative triangularity of −0.8. **d**, Snowflake configuration with a time-varying control of the bottom X-point, where the target X-points are marked in blue. Extended traces for these shots can be found in Extended Data Fig. 2. Source data

**Extended Data Fig. 1:**
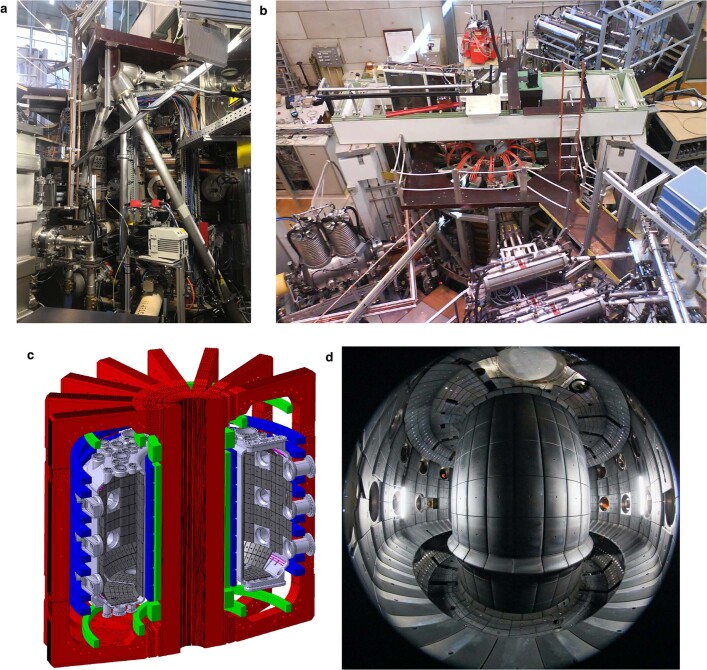
Pictures and illustration of the TCV. **a, b **Photographs showing the part of the TCV inside the bioshield. **c** CAD drawing of the vessel and coils of the TCV. **d** View inside the TCV (Alain Herzog/EPFL), showing the limiter tiling, baffles and central column.

We next demonstrate the capability of our architecture to produce complex configurations for scientific study. Each demonstration has its own time-varying targets but, otherwise, uses the same architectural setup to generate a control policy, including the training and environment configuration, with only minor adjustments to the reward function (shown in Extended Data Table 3). Recall that, in each experiment, the plasma has low elongation before the handover, and the control policy actively modulates the plasma to the configuration of interest. Selected time slices from these experiments are shown in Fig. 3, with further detail in Extended Data Fig. 1 and error metrics in Extended Data Table 1.

Elongating plasmas improves their thermal confinement properties, but their increased vertical-instability growth rate complicates control. We targeted a high elongation of 1.9 with a considerable growth rate. The controller was able to produce and stabilize this elongation, as shown in Fig. 3a. We obtained a good match between the targeted and the desired elongation, with an RMSE of 0.018. We also controlled shape and plasma current to their target values, with an *I*_p_ RMSE of 1.2 kA and shape RMSE of 1.6 cm. This demonstrates the capability to stabilize a high vertical-instability growth rate of more than 1.4 kHz, despite acting at only 10 kHz.

We next tested applying auxiliary heating through neutral beam injection to enter ‘H-mode’, which is desirable for having higher energy confinement time, but causes notable changes to the plasma properties. We were provided a time-varying trajectory on the basis of the proposed ITER configuration that uses such auxiliary heating. As the normalized pressure *β*_p_ increases to 1.12, seen in Fig. 3b, the plasma position and current were maintained accurately, with an *I*_p_ RMSE of 2.6 kA and shape RMSE of 1.4 cm. This shows that our controller can robustly adapt to a changing plasma state and can work with heated H-mode plasma under externally specified configurations.

Negative triangularity plasmas are attractive as they have favourable confinement properties without the strong edge pressure gradient typical of H-modes. We targeted a diverted configuration with triangularity of −0.8, and with X-points at both corners. We successfully achieved this configuration, shown in Fig. 3c. The triangularity was accurately matched, with an RMSE of 0.070, as were the plasma current and shape, with RMSE values of 3.5 kA and 1.3 cm, respectively. This demonstrates the ability to rapidly and directly create a configuration under active study^27^.

Snowflake configurations are researched^28,29^, as they distribute the particle exhaust across several strike points. A crucial parameter is the distance between the two X-points that form the divertor legs. We demonstrated our ability to control this distance, shown in Fig. 3d. The control policy first established a snowflake configuration with X-points separated by 34 cm. It then manipulated the far X-point to approach the limiting X-point, ending with a separation of 6.6 cm. The time-varying X-point targets were tracked with a combined RMSE of 3.7 cm. The plasma current and shape were maintained to high accuracy during this transition, with RMSE values of 0.50 kA and 0.65 cm, respectively. This demonstrates accurate control of a complex time-varying target with several coupled objectives.

In aggregate, these experiments demonstrate the ease with which new configurations can be explored, prove the ability of our architecture to operate in high-performance discharges and confirm the breadth of its capability. In the Methods section, we further investigate the control-policy behaviours.

## New multi-domain plasma demonstration

**Fig. 4:**
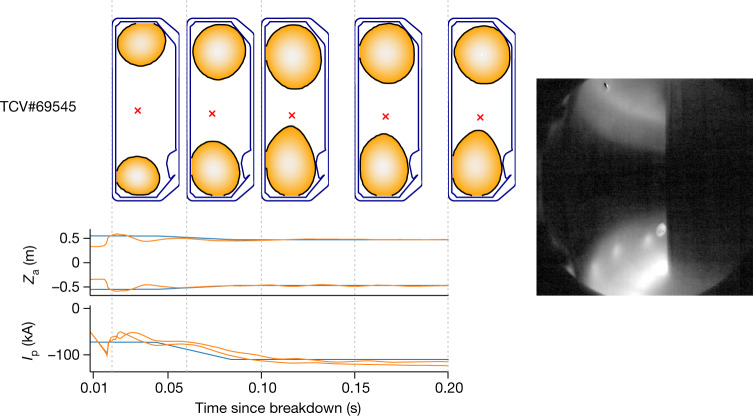
Droplets. Demonstration of sustained control of two independent droplets on TCV for the entire 200-ms control window. Left, control of *I*_p_ for each independent lobe up to the same target value. Right, a picture in which the two droplets are visible, taken from a camera looking into the vessel at *t* = 0.55. Source data

Lastly, we demonstrate the power of our architecture to explore new plasma configurations. We test control of ‘droplets’, a configuration in which two separate plasmas exist within the vessel simultaneously. It is probably possible that existing approaches could stabilize such droplets. Nonetheless, great investment would be required to develop feedforward coil-current programming, implement real-time estimators, tune controller gains and successfully take control after plasma creation. By contrast, with our approach, we simply adjust the simulated handover state to account for the different handover condition from single-axis plasmas and define a reward function to keep the position of each droplet component steady while ramping up the domain plasma currents. This loose specification gives the architecture the freedom to choose how to best adapt the droplet shapes as *I*_p_ increases to maintain stability. The architecture was able to successfully stabilize droplets over the entire 200 ms control window and ramp the current within each domain, as shown in Fig. 4. This highlights the advantage of a general, learning-based control architecture to adapt control for previously unknown configurations.

## Discussion

We present a new paradigm for plasma magnetic confinement on tokamaks. Our control design fulfils many of the hopes of the community for a machine-learning-based control approach^14^, including high performance, robustness to uncertain operating conditions, intuitive target specification and unprecedented versatility. This achievement required overcoming gaps in capability and infrastructure through scientific and engineering advances: an accurate, numerically robust simulator; an informed trade-off between simulation accuracy and computational complexity; a sensor and actuator model tuned to specific hardware control; realistic variation of operating conditions during training; a highly data-efficient RL algorithm that scales to high-dimensional problems; an asymmetric learning setup with an expressive critic but fast-to-evaluate policy; a process for compiling neural networks into real-time-capable code and deployment on a tokamak digital control system. This resulted in successful hardware experiments that demonstrate fundamental capability alongside advanced shape control without requiring fine-tuning on the plant. It additionally shows that a free-boundary equilibrium evolution model has sufficient fidelity to develop transferable controllers, offering a justification for using this approach to test control of future devices.

Efforts could further develop our architecture to quantify its robustness through analysis of the non-linear dynamics^30–32^ and reduce training time through increased reuse of data and multi-fidelity learning^33^. Additionally, the set of control targets can be expanded, for example, to reduce target heat loads through flux expansion^5^, aided by the use ofprivileged information in the critic to avoid requiring real-time observers. The architecture can be coupled to a more capable simulator, for example, incorporating plasma pressure and current-density-evolution physics, to optimize the global plasma performance.

Our learning framework has the potential to shape future fusion research and tokamak development. Underspecified objectives can find configurations that maximize a desired performance objective or even maximize power production. Our architecture can be rapidly deployed on a new tokamak without the need to design and commission the complex system of controllers deployed today, and evaluate proposed designs before they are constructed. More broadly, our approach may enable the discovery of new reactor designs by jointly optimizing the plasma shape, sensing, actuation, wall design, heat load and magnetic controller to maximize overall performance.

## Methods

### Tokamak à Configuration Variable

The TCV ^1,34^, shown in Fig. 1, is a research tokamak at the Swiss Plasma Center, with a major radius of 0.88 m and vessel height and width of 1.50 m and 0.512 m, respectively. TCV has a flexible set of magnetic coils that enable the creation of a wide range of plasma configurations. Electron cyclotron resonance heating and neutral beam injection^35^ systems provide external heating and current drive, as used in the experiment in Fig. 3b. TCV is equipped with several real-time sensors and our control policies use a subset of these sensors. In particular, we use 34 of the wire loops that measure magnetic flux, 38 probes that measure the local magnetic field and 19 measurements of the current in active control coils (augmented with an explicit measure of the difference in current between the ohmic coils). In addition to the magnetic sensors, TCV is equipped with other sensors that are not available in real time, such as the cameras shown in Figs. 2 and 4. Our control policy consumes the magnetic and current sensors of TCV at a 10-kHz control rate. The control policy produces a reference voltage command at each time step for the active control coils.

### Tokamak simulator

The coupled dynamics of the plasma and external active and passiveconductors are modelled with a free-boundary simulator, FGE^22^. The conductors are described by a circuit model in which the resistivity is considered known and constant, and the mutual inductance is computed analytically.

The plasma is assumed to be in a state of toroidally symmetric equilibrium force balance (Grad–Shafranov equation^21^), in which the Lorentz force *J* × *B* generated from the interaction of the plasma current density, *J*, and the magnetic field, *B*, balances the plasma pressure gradient ∇*p*. The transport of radial pressure and current density caused by heat and current drive sources is not modelled. Instead, the plasma radial profiles are modelled as polynomials whose coefficients are constrained by the plasma current *I*_p_ plus two free parameters: the normalized plasma pressure *β*_p_, which is the ratio of kinetic pressure to the magnetic pressure, and the safety factor at the plasma axis *q*_A_, which controls the current density peakedness.

The evolution of the total plasma current *I*_p_, is described as a lumped-parameter equation on the basis of the generalized Ohm’s law for the magnetohydrodynamics model. For this model, the total plasma resistance, *R*_p_, and the total plasma self-inductance, *L*_p_, are free parameters. Finally, FGE produces the synthetic magnetic measurements that simulate the TCV sensors, which are used to learn the control policies, as discussed below.

### Specific settings for the droplets

In the experiment with the droplets (Fig. 4), the plasma is considered pressureless, which simplifies the numerical solution of the force balance equation. Moreover, the G coil was disabled in simulation, as it was placed in open circuit during experiments (the fast radial fields it generates were deemed unnecessary for these plasmas). This experiment used an earlier model for the *I*_p_ evolution designed for stationary-state plasma operation. This model has one free parameter, the radial profile of the neoclassical parallel plasma conductivity ${\sigma }_{\parallel }$ (ref. ^22^). This model was replaced with the one described above for the single-domain plasma experiment, as it better describes the evolution of *I*_p_, especially when it is changing rapidly.

### Plasma parameter variation

We vary the plasma-evolution parameters introduced above during training to provide robust performance across the true but unknown condition of the plasma. The amount of variation is set within ranges identified from experimental data as shown in Extended Data Table 2. In the single-plasma experiments, we vary the plasma resistivity *R*_p_, as well as the profile parameters *β*_p_ and *q*_A_. *L*_p_ is not varied, as it can be computed from a simple relation^36^. These are all independently sampled from a parameter-specific log-uniform distribution. In the experiment with droplets, we vary the initial ohmic coil current values according to a uniform distribution. We set two different values for the droplet ${\sigma }_{\parallel }$ components. We sample the log of the difference between them from a scaled beta distribution and the overall shift in the combined geometric mean from a log-uniform distribution, and then solve for the individual ${\sigma }_{\parallel }$. Parameter values are sampled at the beginning of each episode and kept constant for the duration of the simulation. The sampled value is deliberately not exposed to the learning architecture because it is not directly measureable. Therefore, the agent is forced to learn a controller that can robustly handle all combinations of these parameters. This informed and targeted domain-randomization technique proved to be effective to find policies that track time targets for shape and *I*_p_ while being robust to the injection of external heating and the edge-localized mode perturbations during high confinement mode.

### Sensing and actuation

The raw sensor data on TCV go through a low-pass filtering and signal-conditioning stage^37^. We model this stage in simulation by a time delay and a Gaussian noise model, identified from data during a stationary-plasma operation phase (Extended Data Table 2). This sensor model (shown in Fig. 1b) captures the relevant dynamics affecting control stability. The power-supply dynamics (also shown in Fig. 1b) are modelled with a fixed bias and a fixed time delay identified from data, as well as a further offset varied randomly at the beginning of each episode. The values for these modifications can be found in Extended Data Table 2. This is a conservative approximation of the true thyristor-based power supplies^37^, but captures the essential dynamics for control purposes.

The control policy can learn to be robust against very non-linear hardware-specific phenomena. For example, when the current in the active coils changes polarity and the controller requests a too low voltage, the power supplies can get ‘stuck’, erroneously providing zero output current over an extended period of time (Extended Data Fig. 4b). This phenomenon might affect both the controller stability and the precision. To demonstrate the capability of our controller to deal with this issue, we applied ‘learned-region avoidance’ in the advanced control demonstration to indicate that currents near zero are undesirable. As a result, the control policy effectively learns to increase the voltages when changing the current polarity to avoid stuck coils on the plant (Extended Data Fig. 4c).

### Neural-network architecture

MPO^23^ uses two neural-network architectures to design and optimize the policy: the critic network and the policy network. Both networks are adapted during training, but only the policy network is deployed on the plant.

For the critic network, the inputs are combined with the hyperbolic tangent function value of the last commanded action and fed to a long short-term memory (LSTM) layer 256 units wide. The outputs of the LSTM layer are then concatenated with its inputs and fed to a multilayer perceptron (MLP), that is, a stack of two densely connected hidden layers with 256 latents each. Each of the MLP layers uses an exponential linear unit non-linearity. Finally, we use a last linear layer to output the Q-value.

The policy network is restricted to a network architecture that can be evaluated on the target hardware within 50 μs to obtain the necessary 10-kHz control rate. Additionally, the network needs to perform this inference to sufficient numerical accuracy on the control system, which uses a different processor architecture from the hardware used for training. Therefore, the policy network is built as follows. We feed the inputs to a stack of a linear layer with 256 outputs. The outputs of this linear layer are normalized with a LayerNorm^38^ and bounded using a hyperbolic tangent function. After this, the output is fed through a three-layer MLP using exponential linear unit non-linearity and 256 latents each. The output of this stack is fed through a final linear layer that outputs two parameters per action: one mean of the Gaussian distribution and one standard deviation of the Gaussian distribution. The standard deviation uses a softplus non-linearity to make sure it is always positive. The parameters of this Gaussian distribution over actions are the output of the neural network. Note that, for assessing the policy in simulation and executing on TCV, only the mean of the distribution is used. With this small neural network, we can perform inference within the L2 cache of the CPU on the control system.

These neural networks are initialized with the weights of a truncated normal distribution scaled with the number of inputs and a bias of zero. The exception is the last layer of the policy network, which is initialized the same way but scaled with 0.0001 (ref. ^39^). These networks are trained with an unroll length of 64 steps. For training, we used a batch size of 256 and a discount of 0.99.

Extended Data Figure 5a shows the importance of an asymmetric design between the actor network and the critic network. We compare the standard setup with a symmetric setup in which the critic is also limited by the control rate on the plant. In the standard setup, the critic network is much larger than the policy network (718,337 parameters compared with 266,280 parameters) and also uses a recurrent LSTM. In the symmetric setup, the critic is also an MLP that is about the same size as the policy (266,497 parameters). We see that the symmetric design notably underperforms the asymmetric design in learning an effective policy. We additionally find that the main benefit comes from the recurrent design in the critic to handle the non-Markovian properties of this environment. When we scale up the critic keeping the feedforward structure of the policy, we find that widening its width to 512 units (926,209 parameters) or even 1,024 units (3,425,281 parameters) still does not match the performance of the setup with the smaller but recurrent critic.

### Learning loop

Our approach uses an episodic training approach in which data are collected by running the simulator with a control policy in the loop, as shown in Fig. 1a. The data from these interactions are collected in a finite-capacity first-in-first-out buffer^40^. The interaction trajectories are sampled at random from the buffer by a ‘learner’, which executes the MPO algorithm to update the control-policy parameters. During training, the executed control policy is stochastic to explore successful control options. This stochastic policy is represented by a diagonal Gaussian distribution over coil actions.

**Extended Data Fig. 2:**
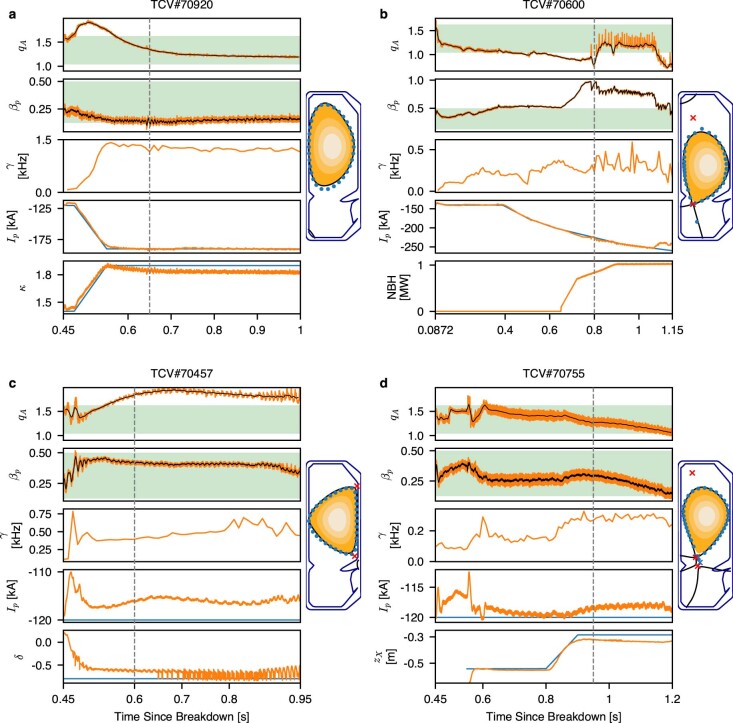
A larger overview of the shots in Fig. 3. We plotted the reconstructed values for the normalized pressure *β*_p_ and safety factor *q*_A_, along with the range of domain randomization these variables saw during training (in green), which can be found in Extended Data Table 2. We also plot the growth rate, *γ*, and the plasma current, *I*_p_, along with the associated target value. Where relevant, we plot the elongation *κ*, the neutral beam heating, the triangularity *δ* and the vertical position of the bottom X-point *Z*_X_ and its target. Source data

Each episode corresponds to a single simulation run that terminates either when a termination condition is hit, which we will discuss below, or when a fixed simulation time has passed in the episode. This fixed time was 0.2 s for the droplets, 0.5 s in the case of Extended Data Fig. 2a, c, and 1 s otherwise. Each episode is initialized from an equilibrium state at the preprogrammed handover time, which was reconstructed from a previous experiment on TCV.

Our training loop emulates the control frequency of 10 kHz. At each step, the policy is evaluated using the observation from the previous step. The resulting action is then applied to the simulator, which is then stepped. Observations and rewards are also collected at the 10-kHz control frequency, resulting in training data collected at 0.1 ms intervals. For our simulation, we chose a time step of 50 kHz. Hence, for each evaluation of the policy, five simulation time steps are computed. The action, that is, the desired coil voltage, is kept constant during these substeps. Data from intermediate steps are only used for checking termination conditions and are discarded afterwards. This enables choosing the control rate and simulator time step independently and, hence, setting the latter on the basis of numerical considerations.

We use a distributed architecture^41^ with a single learner instance on a tensor processing unit and several actors each running an independent instance of the simulator. We used 5,000 actors in parallel for our experiments, generally resulting in training times of 1-3 days, although sometimes longer for complex target specifications. We ran a sweep on the number of actors required to stabilize a basic plasma and the results can be seen in Extended Data Fig. 5. We see that a similar level of performance can be achieved with a large reduction in the number of actors for a moderate cost in training time.

As RL only interacts sample-wise with the environment, the policy could be fine-tuned further with data from interacting with the plant. Alternatively, one might imagine leveraging the database of past experiments performed on TCV to improve the policy. However, it is unclear if the data are sufficiently diverse, given the versatility of TCV and the fact that the same plasma configuration can be achieved by various coil-voltage configurations. Especially for previously unknown plasma shapes, no data or only very limited data are available, rendering this approach ineffective. Conversely, the simulator can directly model the dynamics for the configurations of interest. This issue in which data collection requires a good policy becomes even more pronounced if one wants to optimize a policy de novo from data, without relying on a simulator model.

### Rewards and terminations

All of our experiments have several objectives that must be satisfied simultaneously. These objectives are specified as individual reward components that track an aspect of the simulation — typically, a physical quantity — and these individual components are combined into a single scalar reward value. Descriptions of the targets used are listed in Extended Data Table 4. The target values of the objectives are often time-varying (for example, the plasma current and boundary target points), and are sent to the policy as part of the observations. This time-varying trace of targets is defined by a sequence of values at points in time, which are linearly interpolated for all time steps in between.

Shape targets for each experiment were generated using the shape generator^42^ or specified manually. These points are then canonicalized to 32 equally spaced points along a spline, which are the targets that are fed to the policy. The spline is periodic for closed shapes but non-periodic for diverted shapes, ending at the X-points.

The process for combining these multiple objectives into a single scalar is as follows. First, for each objective, the difference between the actual and target values is computed, and then transformed with a non-linear function to a quality measure between 0 and 1. In the case of a vector-valued objective (for example, distance to each target-shape point), the individual differences are first merged into a single scalar through a ‘combiner’, a weighted non-linear function. Finally, a weighted combination of the individual objective-specific quality measures is computed into a single scalar reward value between 0 and 1 using a combiner as above. This (stepwise) reward is then normalized so that the maximum cumulative reward is 100 for 1 s of control. In cases in which the control policy has triggered a termination, a large negative reward is given. See Extended Data Table 5 for more details.

We typically compute the quality measure from the error using a softplus or sigmoid, which provides a non-zero learning signal early in training when the errors are large, while simultaneously encouraging precision as the policy improves. Similarly, we combine the rewards using a (weighted) smooth max or geometric mean, which gives a larger gradient to improving the worst reward, while still encouraging improving all objectives. The precise reward definitions used in each of our experiments are listed in Extended Data Table 3 and the implementations are available in the supplementary material.

### Further findings

Some controllers exhibited several interesting behaviours, which are briefly mentioned here. These control behaviours hint at further potential capabilities of learned-control approaches.

**Extended Data Fig. 3:**
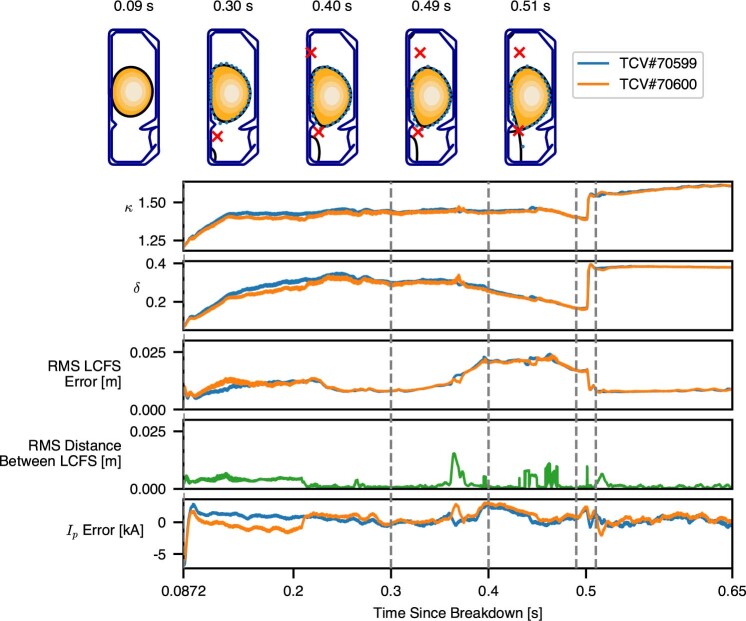
Control variability. To illustrate the variability of the performance that our deterministic controller achieves on the environment, we have plotted the trajectories of one policy that was used twice on the plant: in shot 70599 (in blue) and shot 70600 (in orange). The dotted line shows where the cross sections of the vessel are illustrated. The trajectories are shown from the handover at 0.0872 s until 0.65 s after the breakdown, after which, on shot 70600, the neutral beam heating was turned on and the two shots diverge. The green line shows the RMSE distance between the LCFS in the two experiments, providing a direct measure of the shape similarity between the two shots. This illustrates the repeatability of experiments both in shape parameters such as elongation *κ* and triangularity *δ* and in the error achieved with respect to the targets in plasma current *I*_p_ and the shape of the last closed-flux surface. Source data

External heating was applied during the experiment shown in Fig. 3b. We first ran a test experiment without heating, but with the exact same controller and objectives. This provides a simple repeatability test in the control window before heating was applied. A performance comparison is depicted in Extended Data Fig. 3 and shows that, in these two experiments, the controller performed similarly.

**Extended Data Fig. 4:**
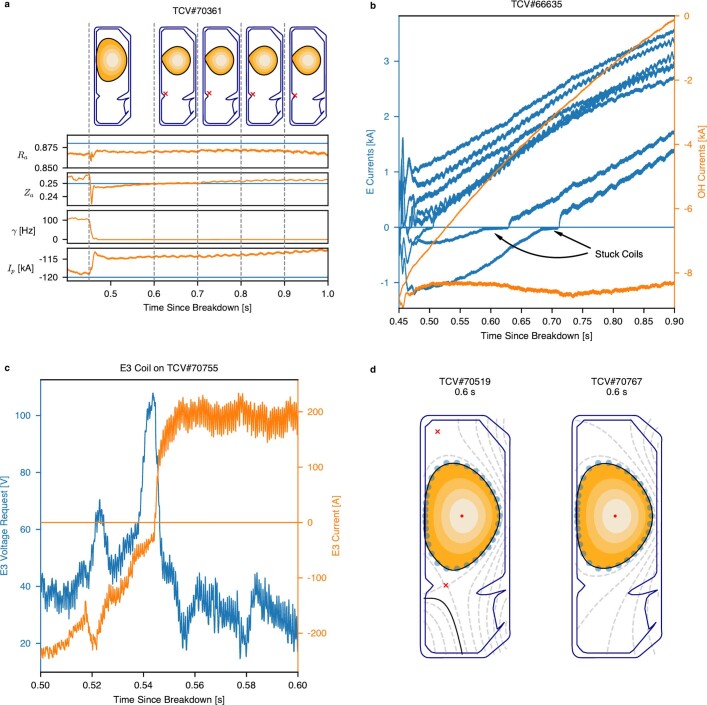
Further observations. **a**, When asked to stabilize the plasma without further specifications, the agent creates a round shape. The agent is in control from *t* = 0.45 and changes the shape while trying to attain *R*_a_ and *Z*_a_ targets. This discovered behaviour is indeed a good solution, as this round plasma is intrinsically stable with a growth rate *γ* < 0. **b**, When not given a reward to have similar current on both ohmic coils, the algorithm tended to use the E coils to obtain the same effect as the OH001 coil. This is indeed possible, as can be seen by the coil positions in Fig. 1g, but causes electromagnetic forces on the machine structures. Therefore, in later shots, a reward was added to keep the current in both ohmic coils close together. **c**, Voltage requests by the policy to avoid the E3 coil from sticking when crossing 0 A. As can be seen in, for example, Extended Data Fig. 4b, the currents can get stuck on 0 A for low voltage requests, a consequence of how these requests are handled by the power system. As this behaviour was hard to model, we introduced a reward to keep the coil currents away from 0 A. The control policy produces a high voltage request to move through this region quickly. **d**, An illustration of the difference in cross sections between two different shots, in which the only difference is that the policy on the right was trained with a further reward for avoiding X-points in vacuum. Source data

When given the goal to maintain only the plasma position and current, our architecture autonomously constructed a low-elongation plasma that eliminates the vertical instability mode (Extended Data Fig. 4a), without being explicitly told to do so.

Our control architecture can naturally choose to use a varying combination of poloidal field and ohmic coils to drive the inductive voltage required for sustaining the plasma current (Extended Data Fig. 4b), in contrast to existing control architectures that typically assume a strict separation.

Our architecture can learn to include non-linear physical and control requests by adding objectives to the goal specification. It can, for example, avoid limitations in the power supplies that occasionally cause ‘stuck’ control-coil currents when reversing polarity (Extended Data Fig. 4c) and avoid X-points in the vessel but outside the plasma (Extended Data Fig. 4d) when requested with high-level rewards.

We see that, for some quantities, there is a steady-state error in the target value (for example, *κ* in Extended Data Fig. 3). Future development will be towards removing such errors, for example, by making the control policy recurrent rather than feedforward. Care must be taken to ensure that these more powerful recurrent policies do not overspecialize to the specific dynamics of the simulator and continue to transfer to TCV successfully.

### Deployment

As the stochastic nature of the training policy is only useful for exploration, the final control policy is taken to be the mean of the Gaussian policy at the conclusion of training. This gives a deterministic policy to execute on the plant. During training, we monitor the quality of this deterministic policy before deployment.

The control loop of TCV runs at 10 kHz, although only half of the cycle time, that is, 50 μs, is available for the control algorithm due to other signal processing and logging. Therefore we created a deployment system that compiles our neural network into real-time-capable code that is guaranteed to run within this time window. To achieve this, we remove superfluous weights and computations (such as the exploration variance) and then use tfcompile^43^ to compile it into binary code, carefully avoiding unnecessary dependencies. We tailored the neural network structure to optimize the use of the processor’s cache and enable vectorized instructions for optimal performance. The table of time-varying control targets is also compiled into the binary for ease of deployment. In future work, targets could easily be supplied at runtime to dynamically adjust the behaviour of the control policy. We then test all compiled policies in an automated, extensive benchmark before deployment to ensure that timings are met consistently.

### Post-experiment analysis

The plasma shape and position are not directly observed and need to be inferred from the available magnetic measurements. This is done with magnetic-equilibrium reconstruction, which solves an inverse problem to find the plasma-current distribution that respects the force balance (Grad–Shafranov equation) and best matches the given experimental magnetic measurements at a specific time in a least-squares sense.

In a conventional magnetic control design, a real-time-capable magnetic-equilibrium reconstruction is needed as a plasma-shape observer to close the shape-control feedback loop (shown as the ‘Plasma shape’ observer in Fig. 1f). In our approach, instead, we only make use of equilibrium reconstruction with LIUQE code^10^ during post-discharge analysis to validate the plasma-shape controller performances and compute the physical initial conditions for the simulation during training.

After running the experiment, we use this equilibrium-reconstruction code to obtain an estimate of the plasma state and magnetic flux field. Using this approach is consistent with previous literature for evaluating performance^9,10^.

The plasma boundary is defined by the last closed-flux surface (LCFS) in the domain. We extract the LCFS as 32 equiangular points around the plasma axis and then canonicalize with splines to 128 equidistant points. The error distance is computed using the shortest distance between each of the points that defined the target shape and the polygon defined by the 128 points on the LCFS. The shape RMSE is computed across these 32 error distances over all time steps in the time range of interest.

Errors on scalar quantities, such as *I*_p_ or elongation, are computed from the error between the reference and the respective estimation from the equilibrium reconstruction over the time period of interest. The estimate of the growth rate of the vertical displacement instability^6^ is computed from a spectral decomposition of the linearized system of equations of the simulator around the reconstructed equilibrium.

### Comparison with previous work

In recent years, advanced control techniques have been applied to magnetic confinement control. De Tommasi et al.^44^ describe a model-based control approach for plasma-position control using a linear model and a cascaded feedback-control structure. Gerkšič and De Tommasi^45^ propose a model predictive control approach, demonstrating linear model predictive control for plasma position and shape control in simulation, including a feasibility estimate for hardware deployment. Boncagni et al.^46^ have proposed a switching controller, improving on plasma-current tracking on hardware but without demonstrating further capabilities. There has been other previous work in which RL has learned on plasma models, for example, to control the safety factor^47^ or to control the ion-temperature gradient^48^. Recently, Seo et al.^49^ have developed feedforward signals for beta control using RL, which have then been verified on the KSTAR tokamak.

More generally, machine-learning-based approaches are being developed for magnetic-confinement control and fusion in general, not limited to control. A survey of this area is provided by Humphreys et al.^14^, who categorized approaches into seven Priority Research Opportunities, including accelerating science, diagnostics, model extraction, control, large data, prediction and platform development. Early use of neural networks in a control loop for plasma control is presented by Bishop et al.^15^, who used a small-scale neural network to estimate the plasma position and low-dimensional shape parameters, which were subsequently used as error signals for feedback control.

Our architecture constitutes an important step forward in terms of generality, in which a single framework is used to solve a broad variety of fusion-control challenges, satisfying several of the key promises of machine learning and artificial intelligence for fusion set out in ref. ^14^.

### Application to alternative tokamaks

Our approach has been successfully demonstrated on TCV, and we are confident that, with a few basic modifications, our approach is directly applicable to other tokamaks that meet some assumptions and technical requirements laid out below. All present-day tokamaks have been confirmed to respect, from the magnetic control point of view, the coupled equations solved by free-boundary simulators. Equilibrium controllers have routinely been designed on the basis of these models, and — for future tokamaks — there is no reason as of yet to believe this model will no longer be valid. Naturally, we cannot predict the performance of our approach on other kinds of devices.

To simulate a different device, the free-boundary simulator parameters will need to be set appropriately. This includes the machine description with the locations and electrical properties of coils, vessel and limiter, the actuator and sensor characteristics, such as current and voltage ranges, noise and delay. Operational conditions such as the expected range of variation of profile parameters also need to be determined. Finally, rewards and targets need to be updated to match the geometry and desired shapes.

The aforementioned characteristics should be readily available, as these are typically part of the design process for a given tokamak. Indeed, Grad–Shafranov equilibrium calculations are routinely carried out for the general design and analysis of a new tokamak, and these include all required parameters. These variations in vessel geometry and the number, placement and range of sensors and coils should not require changes to the learning algorithm beyond adjusting design bounds. The learning algorithm will automatically adjust input and output layer dimensions for the neural network and will automatically learn a policy suited to the new vessel and control system.

Further considerations are required for deployment. Our approach requires a centralized control system with sufficient computational power to evaluate a neural network at the desired control frequency, although a desktop-grade CPU is sufficient to meet this requirement. Also, an existing magnetic controller is needed to perform plasma breakdown and early ramp-up before handing over to the learned controller. Although our controllers are trained to avoid terminations in simulation corresponding to disruption criteria, they are not guaranteed to avoid plasma disruptions. Hence, if the target tokamak cannot tolerate certain kinds of disruptions, a machine-protection layer such as a simpler fallback controller or interlock system should be in place during experiments.

## Online content

Any methods, additional references, Nature Research reporting summaries, source data, extended data, supplementary information, acknowledgements, peer review information; details of author contributions and competing interests; and statements of data and code availability are available at 10.1038/s41586-021-04301-9.

### Supplementary information

Supplementary Information This file contains an overview of the files located in the accompanying zipped Supplementary Data folder.

Supplementary Data

### Source data

Source Data Fig. 2

Source Data Fig. 3

Source Data Fig. 4

Source Data Extended Data Fig. 2

Source Data Extended Data Fig. 3

Source Data Extended Data Fig. 4

**Extended Data Fig. 5:**
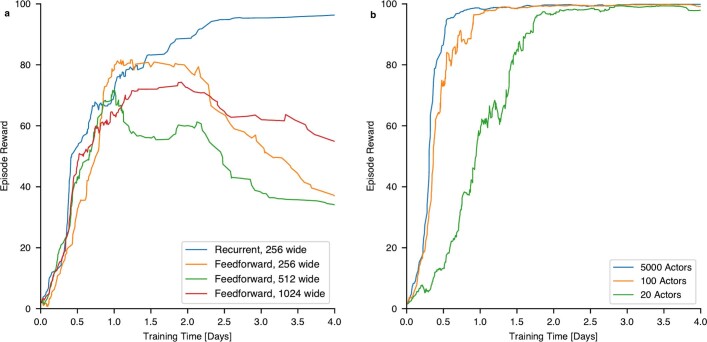
Training progress. Episodic reward for the deterministic policy smoothed across 20 episodes with parameter variations enabled, in which 100 means that all objectives are perfectly met.  **a** comparison of the learning curve for the capability benchmark (as shown in Fig. 2) using our asymmetric actor-critic versus a symmetric actor-critic, in which the critic is using the same real-time-capable feedforward network as the actor. In blue is the performance with the default critic of 718,337 parameters. In orange, we show the symmetric version, in which the critic has the same feedforward structure and size (266,497 parameters) as the policy (266,280 parameters). When we keep the feedforward structure of the symmetric critic and scale up the critic, we find that widening its width to 512 units (in green, 926,209 parameters) or even 1,024 units (in red, 3,425,281 parameters) does not bridge the performance gap with the smaller recurrent critic. **b** comparison between using various amounts of actors for stabilizing a mildly elongated plasma. Although the policies in this paper were trained with 5,000 actors, this comparison shows that, at least for simpler cases, the same level of performance can be achieved with much lower computational resources. Source data

Source Data Extended Data Fig. 5

## Figures and Tables

**Extended Data Table 1:**
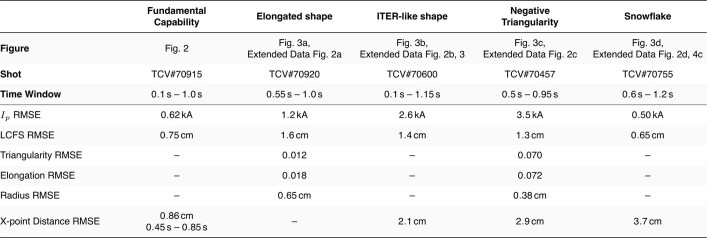
Performance metrics of experiments

Various performance metrics of the shots on the plant are tabulated here. The triangularity, elongation and radius are only shown for policies in which this was a target measure. The X-point distance is only computed in the time window where an X-point target was requested.

**Extended Data Table 2:**
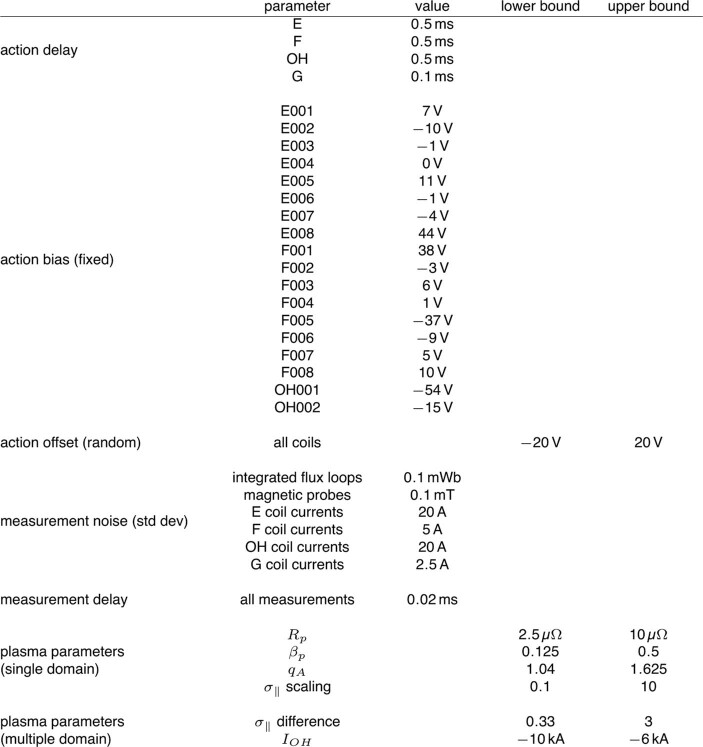
Simulation parameters for actuator, sensor and current diffusion models

Parameter values as identified from data. The action bias was fit on the power supply output voltage. Measurement noise is Gaussian additive noise and randomly sampled at each simulation time step. We use a fixed action bias with an additive random offset to account for non-ideal behaviour of power supply hardware. Current diffusion-parameter variations account for the uncontrolled operating conditions. Parameter variations are sampled at the beginning of each episode but kept constant during the episode. The samples are drawn from uniform (action bias) and log-uniform (current diffusion) distributions using the bounds in this table. For single-plasma training, *R*_p_, *β*_p_ and *q*_A_ are varied, whereas in a multiple-plasmas training, we vary ${\sigma }_{\parallel }$ and *I*_OH_. In the latter case, we sample an overall geometric mean offset of the two ${\sigma }_{\parallel }$ from a log-uniform distribution. We sample the log of the multiplicative difference between them from *B*_s_ (4,4), for which *B*_s_ is a scaled *β* distribution. We sample a single *I*_OH_ value for both coils. Parameters are sampled as absolute values unless explicitly indicated as scaling factors.

**Extended Data Table 3:**
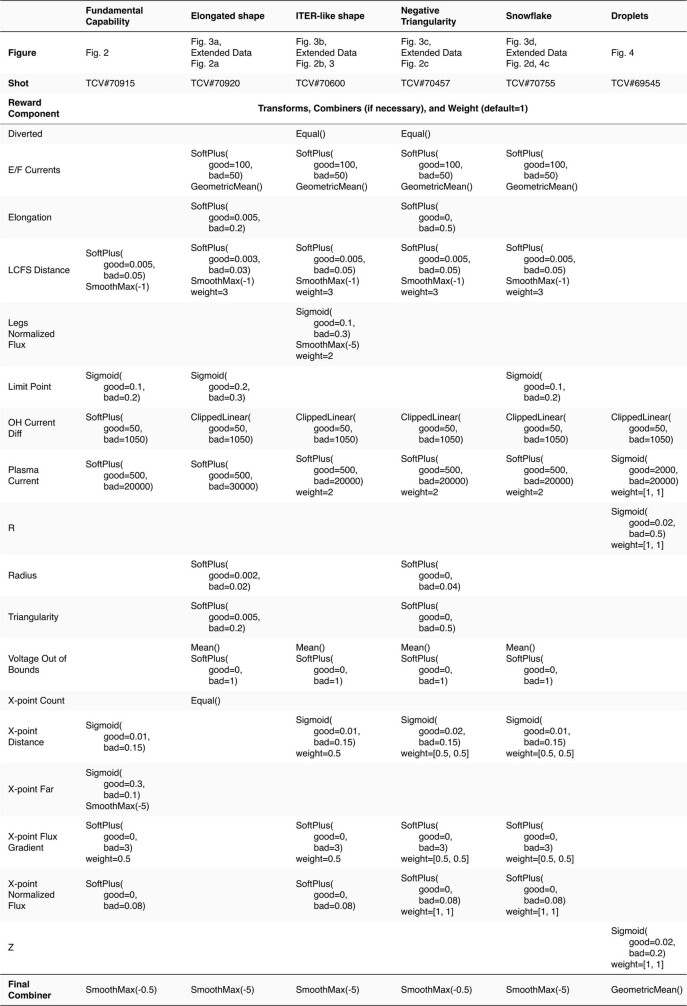
Rewards used in the experiments

Empty cells are not used in that reward. Any cell that does not specify a weight has an implicit weight of 1. Vector-valued weights (for example, Droplets: R) return several values to the final combiner. See Exended Data Table 4 for the descriptions of the different reward components and Extended Data Table 5 for the transforms, combiners and terminations. All of the terminations criteria were used for these experiments. Code for these rewards is available in the supplementary material.

**Extended Data Table 4:**
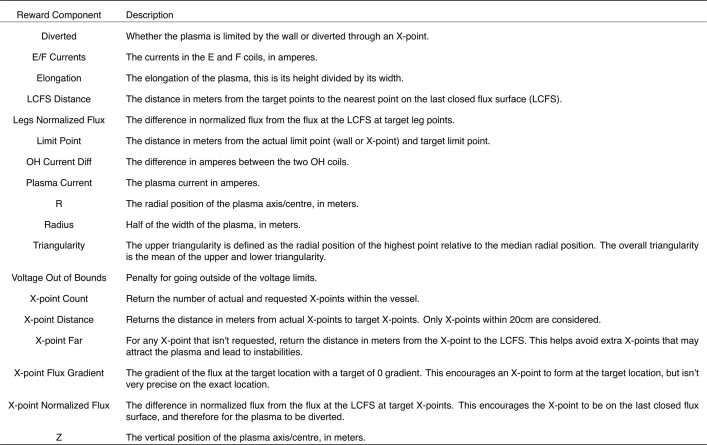
Reward components

Description of reward components. All of these return an actual and a target value, and many allow time-varying target values. See Extended Data Table 3 for where and how they are used.

**Extended Data Table 5:**
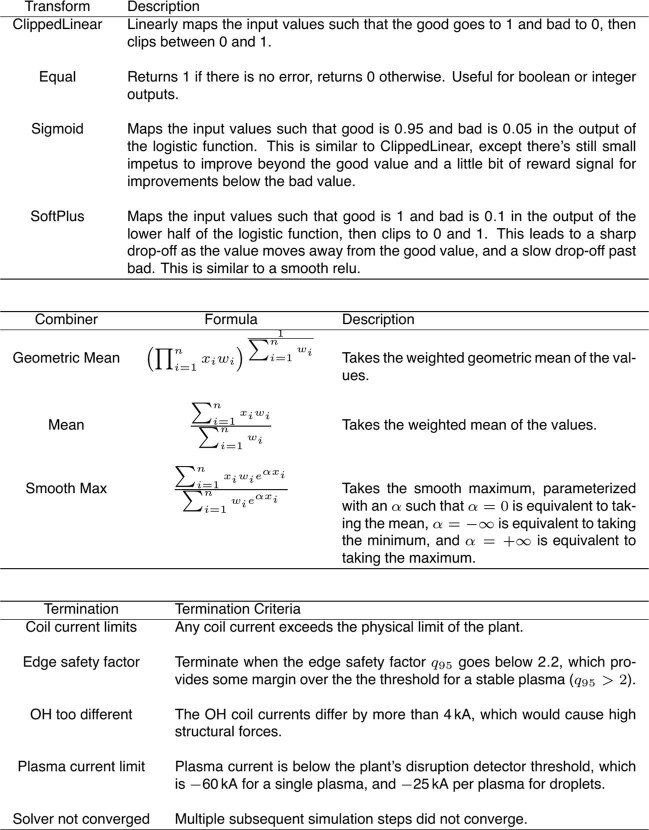
Reward elements

Elements used to construct reward functions. Transforms scale the different reward component. The *q*_95_ value is as  defined^54^. Transforms take a good and bad value that usually have some semantic meaning defined by the reward component and then map it to the range 0-1. The good value should lead to a reward close or equal to 1, whereas a bad value should lead to a reward close or equal to 0. Combiners take a list of values and corresponding weights and return a single value. Any values with a weight of 0 are excluded. Terminations trigger the end of an episode with a large negative reward. Specific implementations are in the Supplementary Data.

## Data Availability

TCV experimental data from the images in this paper are available in the Supplementary information. Source data are provided with this paper.

## Code availability

The learning algorithm used in the actor-critic RL method is MPO^23^, a reference implementation of which is available under an open-source license^41^. Additionally, the software libraries launchpad^50^, dm_env^51^, sonnet^52^, tensorflow^53^ and reverb^40^ were used, which are also available as open source. The code to compute the control targets, rewards and terminations is available in the Supplementary information. FGE and LIUQE are available subject to license agreement from the Swiss Plasma Center at EPFL (Antoine Merle antoine.merle@epfl.ch, Federico Felici federico.felici@epfl.ch).
