# Supplementary material for: Magnetic control of tokamak plasmas through deep reinforcement learning
Source: Nature. 2022 Feb 16;602(7897):414–9. doi: 10.1038/s41586-021-04301-9 (PMC8850200; doi:10.1038/s41586-021-04301-9)
Supplement: Supplementary file 1 — This file contains an overview of the files located in the accompanying zipped Supplementary Data folder. [file 41586_2021_4301_MOESM1_ESM.pdf]

nature portfolio

https://doi.org/10.1038/s41586-021-04301-9

Supplementary information

Magnetic control of tokamak plasmas through deep reinforcement learning

In the format provided by the authors and unedited

TCV Fusion Control Objectives

This code release contains the rewards, control targets, noise model and parameter variation for the paper *Magnetic Control of Tokamak Plasmas through Deep Reinforcement Learning*.

Further sensor and reconstruction data exceeding the size limits of the Supplementary Materials, are available on the DeepMind repository on github.

Disclaimer

This release is useful for understanding the details of specific elements of the learning architecture, however it does not contain the simulator (FGE, part of LIUQE), the trained/exported control policies, nor the agent training infrastructure. This release is useful for replicating our results which can be done by assembling all the components, many of which are open source elsewhere. Please see the "Code Availability" statement in the paper for more information.

The learning algorithm we used is MPO, which has an open source reference implementation in Acme. Additionally, the open source software libraries launchpad (code), dm_env, sonnet, tensorflow (code) and reverb (code) were used. FGE and LIUQE are available on request from the Swiss Plasma Center at EPFL (email Federico Felici), subject to license agreement.

Objectives used in published TCV experiments

Take a look at `rewards_used.py` and `references.py` for the rewards and control targets used in the paper.

To print the actual control targets, run:

```
$ python3 -m fusion_tcv.references_main --refs=snowflake
```

Make sure to install the dependencies: `pip install -r fusion_tcv/requirements.txt`.

Overview of files

- `agent.py`: An interface for what a real agent might look like. This mainly exists so the run loop builds.
- `combiners.py`: Defined combiners that combine multiple values into one. Useful for creating a scalar reward from a set of reward components.
- `environment.py`: Augments the FGE simulator to make it an RL environment (parameter variation, reward computation, etc.).
- `experiments.py`: The environment definitions published in the paper.
- `fge_octave.py`: An interface for the simulator. Given that FGE isn't open source, this is a sketch of how you might use it.
- `fge_state.py`: A python interface to the simulator state. Given that FGE isn't open source, this is a sketch and returns fake data.
- `named_array.py`: Makes it easier to interact with numpy arrays by name. Useful for referring to parts of the control targets/references.
- `noise.py`: Adds action and observation noise.
- `param_variation.py`: Defines the physics parameters used by the simulation.
- `ref_gen.py`: The tools to generate control targets per time step.
- `references.py`: The control targets used in the experiments.
- `references_main.py`: Runnable script to output the references to the command line.
- `rewards.py`: Computes all of the reward components and combines them together to create a single scalar reward for the environment.
- `rewards_used.py`: The actual reward definitions used in the experiments.
- `run_loop.py`: An example of interacting with the environment to generate a trajectory to send to the replay buffer. This code is notional as a complete version would require an Agent and a Simulator implementation.
- `targets.py`: The reward components that pull data from the control targets and the simulator state for generating rewards. This depends on FGE, so cannot be run.
- `tcv_common.py`: Defines the physical attributes of the TCV fusion reactor, and how to interact with it.
- `terminations.py`: Defines when the simulation should stop.
- `trajectory.py`: Stores the history of the episode.
- `transforms.py`: Turns error values into normalized values.
